# Glioma surgery in eloquent areas: can we preserve cognition?

**DOI:** 10.1007/s00701-015-2601-7

**Published:** 2015-11-14

**Authors:** Djaina Satoer, Evy Visch-Brink, Clemens Dirven, Arnaud Vincent

**Affiliations:** Department of Neurosurgery, Erasmus MC—University Medical Centre, Wytemaweg 80, Room EE220, 3015 GE Rotterdam, The Netherlands; Centre for Language and Cognition Groningen (CLCG), University of Groningen, Groningen, The Netherlands

**Keywords:** Cognition, Eloquent areas, Glioma surgery, Neuropsychological tests, Systematic review

## Abstract

**Background:**

Cognitive preservation is crucial in glioma surgery, as it is an important aspect of daily life functioning. Several studies claimed that surgery in eloquent areas is possible without causing severe cognitive damage. However, this conclusion was relatively ungrounded due to the lack of extensive neuropsychological testing in homogenous patient groups. In this study, we aimed to elucidate the short-term and long-term effects of glioma surgery on cognition by identifying all studies who conducted neuropsychological tests preoperatively and postoperatively in glioma patients.

**Methods:**

We systematically searched the electronical databases Embase, Medline OvidSP, Web of Science, PsychINFO OvidSP, PubMed, Cochrane, Google Scholar, Scirius and Proquest aimed at cognitive performance in glioma patients preoperatively and postoperatively.

**Results:**

We included 17 studies with tests assessing the cognitive domains: language, memory, attention, executive functions and/or visuospatial abilities. Language was the domain most frequently examined. Immediately postoperatively, all studies except one, found deterioration in one or more cognitive domains. In the longer term (3–6/6–12 months postoperatively), the following tests showed both recovery and deterioration compared with the preoperative level: naming and verbal fluency (language), verbal word learning (memory) and Trailmaking B (executive functions).

**Conclusions:**

Cognitive recovery to the preoperative level after surgery is possible to a certain extent; however, the results are too arbitrary to draw definite conclusions and not all studies investigated all cognitive domains. More studies with longer postoperative follow-up with tests for cognitive change are necessary for a better understanding of the conclusive effects of glioma surgery on cognition.

## Introduction

In The Netherlands, the incidence of newly diagnosed primary brain tumours is 5–7 per 100,000, of which 20 % are low-grade gliomas (LGGs) [28]. LGGs are mostly revealed by epileptic seizures and/or by mild cognitive complaints. LGGs often reside in the so-called “eloquent areas” of the brain. However, due to the slow growth rate of LGGs, i.e. 5 mm per year [36], the brain is supposed to be able to reorganise the functions at risk for impairment (e.g. language or motor) [20, 37]. Therefore severe neurological and/or cognitive disturbances are assumed to be relatively rare. Currently, the “gold standard” treatment for LGG is awake surgery with direct electrocortical stimulation to preserve functions. Recent publications show that, with this technique, maximal resection percentages with minimal neurological deficits can be attained [16]. Currently, the specific effects of glioma surgery on higher cognitive functions, such as language, memory, attention and executive functions, however, are not entirely clear.

There is a vast body of literature with reports on the neurological outcomes of patients operated on for brain diseases, such as meningiomas, cavernomas, ependymomas, metastases and gliomas in eloquent areas [5, 23, 48, 55, 62]. These studies have provided knowledge about the tremendous neural plasticity of the brain during the recovery period after surgical intervention. The general observation is that postoperative cognitive deterioration (such as aphasia) is transient and recovers within 3 months. However, there is no real evidence for this assumption related to cognition. Usually, individual cases were presented but no solid group analyses were conducted [19, 21, 35, 53, 56, 69]. Moreover, the majority of these studies used brief neurological screening tools, such as Mini-Mental State Examination (MMSE) and/or Karnofsky Performance Scale (KPS), or limited language tasks, such as naming [14, 17, 20, 22].

Some neurosurgical studies investigated cognition more thoroughly with extensive tests after diagnosis [6, 44] or after (mixed) surgical treatment (before adjuvant therapy) [1, 12, 13, 15, 26, 30, 52]. They highlighted impairments in language and attention/executive functioning. Their results, however, did not provide insight into the effects of surgery, because cognition was investigated on only one time point, i.e. preoperatively or postoperatively. Other studies also conducted neuropsychological tasks, but heterogeneous tumour treatment was applied, such as stereotactic biopsy, total resection, chemotherapy and/or radiotherapy [33, 39, 67], or heterogeneous tumour groups were taken together for analysis [3, 29, 34, 70].

Several investigators already pointed out the relevance of extensive cognitive testing in glioma patients before surgery with a follow-up [31, 43, 49, 63]. However, detailed complete analyses on the effects of extensive surgery on the main cognitive domains, such as language, memory, attention, executive functions and visuospatial abilities is not standard procedure in patients with eloquent area gliomas.

The aim of this systematic review is to search the literature to identify the current status of short-term and longer-term effects of glioma surgery in eloquent areas on different cognitive functions, language, memory, attention/executive functions and visuo-spatial abilities. As a result, patients can be better prepared for their prognosis and sensitive tasks for cognitive change might be revealed, which is essential information for clinical practice.

## Methods

### Search strategy

Our goal was to identify all publications reporting cognitive status in adult glioma patients before and after surgery until 1 July 2013. A double negation filter on “children” was utilised to minimise the results on paediatric literature. We systematically searched the electronical databases, Embase, Medline OvidSP, Web of Science, PsychINFO OvidSP, PubMed, Cochrane, Google Scholar, Scirius and Proquest (*see*Appendix I, which illustrates the search string).

### Study selection criteria

All titles and abstracts were reviewed by the first author (D.S.). Firstly, irrelevant studies were excluded. Then any study reporting on cognition was included for full-text screening. Subsequently we eliminated studies describing patients treated with biopsy, neurological status, heterogeneous tumours (and metastases) and heterogeneous treatment. Publications included in our study concerned an adult patient population with gliomas treated for extensive surgery in eloquent areas who underwent neuropsychological testing (with standardised tests) both before and after surgery. Difficult cases were discussed with two co-authors (E.V. and C.D.).

## Results

**Fig. 1:**
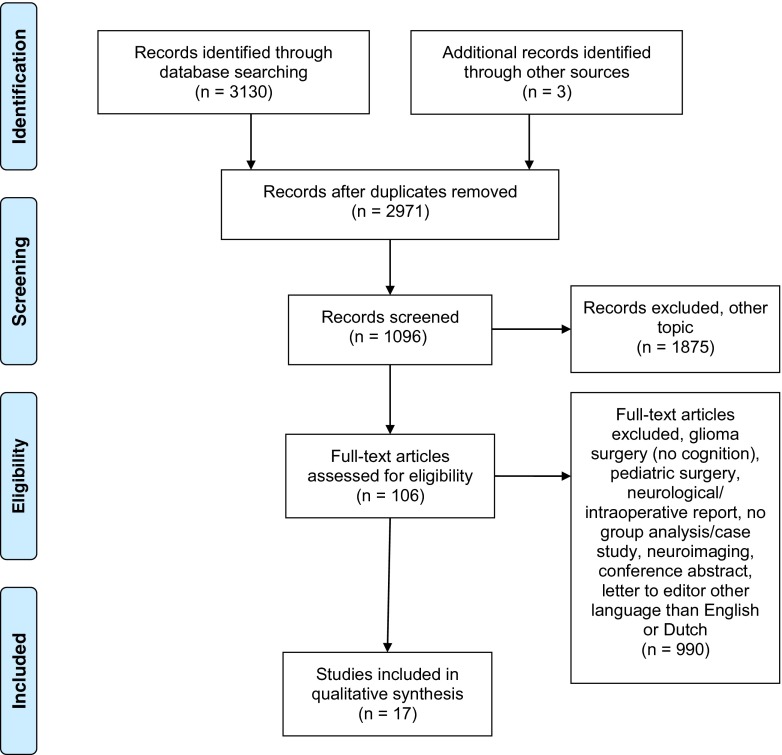
Flowchart search results

The electronic search resulted in 3,130 publications. Three articles were identified by additional “hand-searching” the reference lists (total, 3,133). After title and abstract screening, 162 were duplicates and 1,875 articles were excluded because of irrelevance. Three hundred and fourteen articles discussed glioma surgery, but not cognition or concerned paediatric literature. Six hundred and seventy-six articles were excluded due to: neurological and/or intraoperative reports, no group analysis/case studies, focus on neuroimaging, conference abstract, letter to editor, language other than Dutch or English. One hundred and six full-text publications were evaluated, of which finally 17 articles were selected (*see* Fig. 1).

### Included studies

We identified 17 articles (2006–2013) in which cognitive performance was assessed in glioma patients with an extensive test battery preoperatively and postoperatively, with or without further follow-up. The sample size ranged from 7 [9] to 226 patients (of which a subgroup was analysed) [49]. The interval after tumour resection was different. Nine studies investigated cognition in the immediate postoperative phase, of which seven conducted a follow-up (range, 3 days to 6 months) [4, 8, 9, 45, 50, 57, 64, 66, 73]. Six articles conducted a postoperative examination between 3 months and 12 months [46, 58, 59, 61, 71, 72], and another two designed a prognostic study in which tasks were revealed associated with postoperative relapse in cognition [40, 49]. Eleven studies compared a postoperative follow-up moment to preoperative baseline level [4, 8, 46, 50, 57–59, 61, 66, 71, 72]. Follow-up moments ranged from 1–5 days to 3 years [9, 58]. The most common times of measurement were immediately and 3–6 months postoperatively. Two studies did not report on the exact follow-up time (Sarubbo et al. [58] only mentioned a follow-up of 3 years in the title, but did not provide specifications in the article) nor on specific statistical methods to investigate performance [58, 72]. Five articles discussed cognitive outcome of patients with specific tumour location (e.g. mesial frontal lobe, temporal lobe, insular lobe, uncinate fasciculus, arcuate fasciculus [8, 9, 50, 71, 73]. The remaining studies included patients with gliomas in mixed eloquent areas, i.e. the frontal, temporal, parietal and/or occipital lobes. Table 1 shows details of the studies we identified.

**Table 1:** Study design

Author & year	Surgical intervention	Immediate postoperative testing	Follow-up testing	Tumour grade	*n*
Bello et al. 2007	Awake surgery	Yes	1 month and 3 months	LGG + HGG	88
Teixidor et al. 2007	Awake surgery	Yes	3 months	LGG	23
Yoshii et al. 2008	Awake surgery		Yes, but not clear	LGG^a^ + HGG	31
Chainay et al. 2009	Surgery	Yes	3, 7 days	LGG	7
Campanella et al. 2009	Surgery	Yes	No	LGG + HGG	20
Talacchi et al. 2011	(Sub)total surgery	Yes	No	LGG + HGG	29
Papagno et al. 2011	Awake surgery	Yes	3 months	LGG + HGG	44
Sarubbo et al. 2011	Awake surgery	No	3 years	LGG	12
Wu et al. 2011	Awake surgery	No	Yes, but not clear	LGG + HGG	33
Mattavelli et al. 2012	Awake surgery	Yes	No	LGG	22
Papagno et al. 2012	Awake surgery	Yes	3 months	LGG + HGG	226^b^
Zhao et al. 2012	Awake surgery	Yes	3–6 months	LGG + HGG	20
Santini et al. 2012	Awake surgery	Yes	3–6 months	LGG + HGG	22
Satoer et al. 2012	Surgery	No	3–4 months	LGG + HGG	28
Moritz-Gasser et al. (sub-study 2) 2012	Awake surgery	No	6–12 months	LGG	12
Moritz-Gasser et al. 2013	Awake surgery	Yes	6 months	LGG	8
Satoer et al. 2013	Awake surgery	No	3–4 months	LGG + HGG	27

*LGG* low-grade glioma, *HGG* high-grade glioma

^a^Also meningiomas were included, but this group could be separated from glioma patients in our analysis

^b^At least one follow-up at 3 months was collected for 117 patients

### Neuropsychological protocol

All studies investigated the language domain. Eight studies investigated one or two cognitive domains (including language), and the remaining nine studies examined three to four other different cognitive domains, i.e. memory, attention and executive functions and/or visuo-spatial abilities (or other and including language). The most frequently used tests for assessing language functions concerned: object naming and verbal fluency (category and letter), for memory: verbal word learning (encoding, recall and recognition), verbal/digit span, for attention and executive functions: Trailmaking Test (TMT) A, B. *See* Table 2 for specifics on conducted tasks per domain.

**Table 2:**
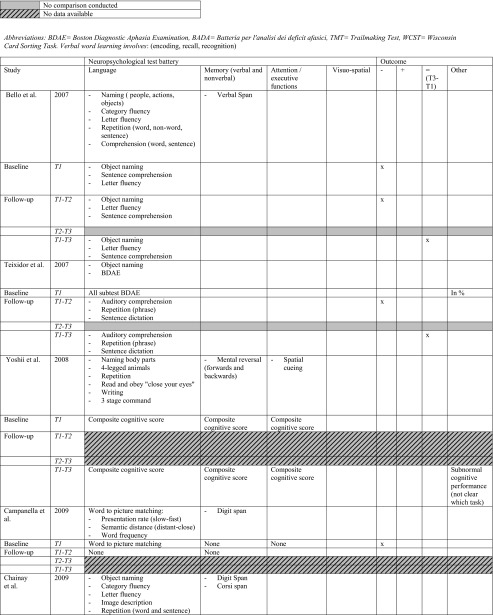

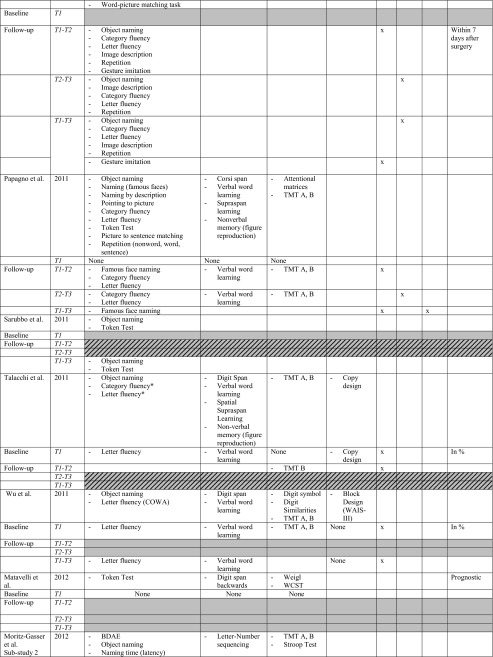

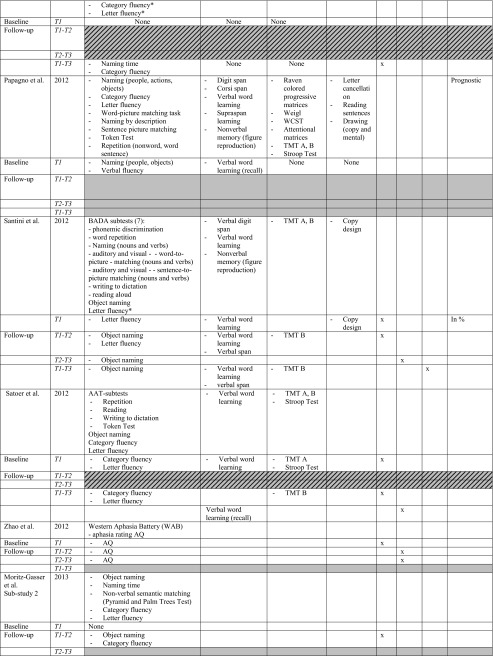

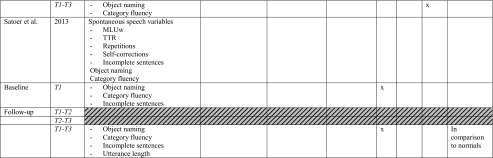
Neuropsychological protocol and outcome. *T1* baseline, *T2* direct postoperatively, *T3* follow-up measurement (*see* Table 1 for specific follow-up period). * Authors categorised fluency task in executive functions. For practical reasons, we classified all fluency tasks in the language domain. −, impairment/decline; +, recovery; =, no difference between test-moments (T3-T1)

*BDAE* Boston diagnostic aphasia examination, *BADA* Batteria per l’analisi dei deficit afasici, *TMT* Trail making test, *WCST* Wisconsin card sorting task. Verbal word learning involves: (encoding, recall, recognition)

### Cognitive baseline and outcome

At preoperative level (T1), eight studies conducted a statistical group analysis compared with a normative group and six provided percentages of impaired performance to indicate impairments [4, 8, 45, 46, 49, 50, 57, 59, 61, 64, 66, 71, 73]. Two studies reported individual scores [40, 58] and one study presented a mean of the tasks without mentioning the normative threshold [45].

The neuropsychological preoperative findings were as follows: language deficits, 12 studies; memory deficits, 3 studies; attention/executive functioning deficits, 3 studies; visuo-spatial domain, 1 study; 1 study mentioned subnormal cognition without specifying the domains/tasks. Only three studies identified no preoperative cognitive deficits [45, 46, 50]. In sum, the majority of the studies found preoperative deficits in one or more cognitive domains.

Nine studies statistically compared immediate postoperative versus preoperative cognitive level (T2-T1) in the following domains: language, all studies; memory, six; attention/executive functions, four; visuospatial abilities, three. In the immediate postoperative phase, seven out of nine studies (78.8 %) found a deterioration in the language domain [4, 8, 9, 45, 50, 57, 66], two out of six (33.3 %) found a decline in the memory domain [50, 57] and three out of four (75 %) in the executive functioning [50, 57, 64]. Only one study found an improvement in the language domain (with Aphasia Quotient) [73].

Six studies investigated the recovery course between the immediate postoperative phase and a follow-up test-moment (T2-T3). Most studies reported no significant difference in performance on tests for language, memory, attention/executive functions or visuo-spatial abilities. Only three studies reported significant improvement in: language (naming [57] verbal fluency [50] and Aphasia Quotient [73]), memory (verbal word learning [57]) and attention/executive functions (TMT A, B [50]).

Eleven studies compared a follow-up test-moment to preoperative level (T3-T1). One study indicated no statistically significant worsening or improvement [58]. In the longer term, five studies reported no significant differences in the language domain between T3-T1 suggesting an improvement to preoperative level of the defective functions in the immediate postoperative phase, in particular in language (naming, verbal fluency, sentence comprehension), but also in memory (verbal word learning), and executive functioning (TMT B) [4, 45, 50, 57, 66]. Six studies, however, still reported a significant cognitive deterioration in one or more domains at follow-up compared with preoperative baseline level, in the domains: language (naming, verbal fluency), memory (verbal word learning) and executive functions (TMT B) [9, 46, 50, 59, 61, 71]. Only one study found a significant improvement in the memory domain (verbal word learning, recall) compared with preoperative baseline level [61].

**Fig. 2:**
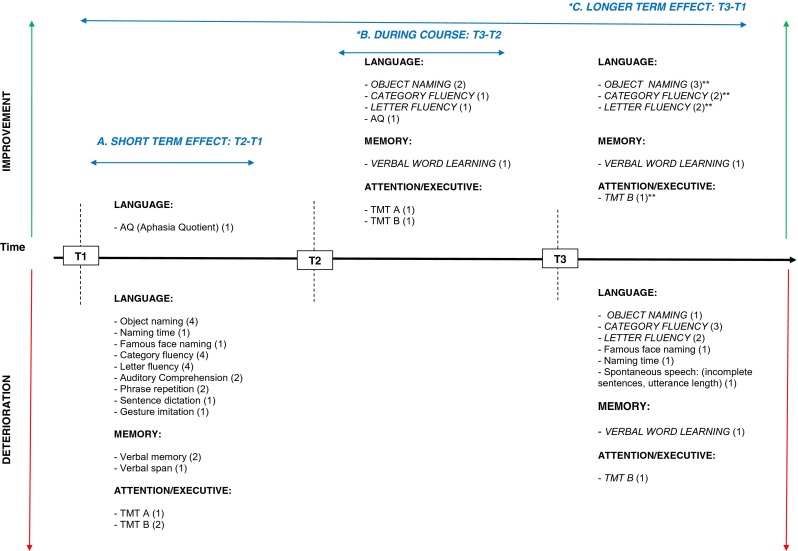
Summary of sensitive neuropsychological tasks for deterioration or improvement in the short and longer term after glioma surgery. *T1* before surgery, *T2* directly after surgery, *T3* follow-up after surgery. *Below the timeline*, a summary is provided of tasks which deteriorated between test moment in the different cognitive domains, whereas improvements are shown *above the timeline*. Comparisons between three different test moments are illustrated: *A* T2-T1, short-term effect of surgery; *B* T2-T1, during course; *C* T3-T1, longer-term effect of surgery. Tasks in *italics* and *capital letters* are tasks that show mixed outcome at short term and/or longer term (3-6 months) after surgery, i.e. they show both deterioration and recovery. The number of studies finding a specific task sensitive for change are presented *in parentheses*. *The sensitive tasks revealed by Chainay et al. (2009) were not considered in this figure as they were all administered within 7 days after surgery. **Some studies reported no significant difference between follow-up phase (*T3*) and preoperative baseline level (*T1*), suggesting recovery at T3 after a decline in the immediate postoperative phase (*T2*): *OBJECT NAMING* (*2*) Bello et al. (2007), Moritz-Gasser et al. (2013); *CATEGORY FLUENCY* (*2*) Moritz-Gasser et al. (2012, 2013); *LETTER FLUENCY* (*2*) Bello et al. (2006), Papagno et al. (2011); *VERBAL MEMORY* (*1*) Papagno et al. (2011); *TMT B* (*1*) Santini et al. (2012)

In short, cognitive disorders in the main cognitive domains are frequently observed preoperatively followed by, for the majority of studies, a decline in the immediate postoperative phase in one or more domains. Language and executive functions seemed to be the most frequently impaired functions direct postoperatively, although also improvement of a general Aphasia Quotient was found. Nearly no significant changes are mentioned between the direct postoperative phase and the follow-up, apart from three studies which found improvement in language, and/or memory and attention/executive functioning [50, 57]. However, compared with the preoperative level, half of the studies mentioned an equal performance whereas deterioration was found in the other studies, apart from an improvement in memory [61]. *See* Table 2 for detailed preoperative cognitive status and postoperative outcome and *see* Fig. 2 for a summary of sensitive tasks short-term postoperatively (T2-T1), during course (T3-T2) and longer-term postoperatively (T3-T1). In addition, overlapping tests with both recovery and deterioration are indicated.

### Tumour characteristics and adjuvant therapy

Eight studies investigated the effect of tumour grade on cognition, of which three pointed out an association between cognitive improvement and high-grade glioma (HGG) [57, 59, 64], whereas one study showed the opposite effect (Table 3) [8].

**Table 3:** Tumour characteristics, adjuvant therapy and cognitive functions

Study	Tumour grade	Volume/EOR	Adjuvant therapy
Bello et al. 2007	No effect	N/A	N/A
Teixidor et al. 2007	N/A	N/A	N/A
Yoshii et al. 2008	No effect	No effect	
Chainay et al. 2009	N/A	N/A	N/A
Campanella et al. 2009	Preop: LH HGG worse than HC and LGG (Word to picture matching, presentation rate + semantic distance)	N/A	N/A
	Postop: RH HGG deteriorates compared to HC		
Talacchi et al. 2011	HGG associated with improvement (word fluency, verbal memory, visuospatial memory, memory domain)	Larger tumour associated with worsening (executive functions, word fluency and TMTB)	N/A
Papagno et al. 2011	No effect	N/A	N/A
Sarubbo et al. 2011	N/A	N/A	N/A
Wu et al. 2011	N/A	N/A	N/A
Matavelli et al. 2012		No effect volume	
Papagno et al. 2012		- Temporal (LH) glioma + volume (covariate) associated with relapse in verbal fluency	
		- Temporal and frontal (LH) + volume (site and grade as covariates) glioma associated with object naming	
Zhao et al. 2012	N/A	N/A	N/A
Santini et al. 2012	HGG associated with improvement	No effect	N/A
Satoer et al. 2012	No effect	No effect	No effect
Moritz-Gasser et al. 2012	N/A	N/A	N/A
Moritz-Gasser et al. 2013	N/A	N/A	N/A
Satoer et al. 2013	LGG worse than HGG and controls in Incomplete sentences	No effect	No effect

*LH* left hemisphere, *RH* right hemisphere, *LGG* low-grade glioma, *HGG* high-grade glioma, *HC* healthy controls, *N/A* not administered (no analysis conducted)

Although all studies reported on tumour location in eloquent areas in either the left or right hemisphere (*see* Table 4), only nine studies statistically examined the relationship between tumour localisation and cognitive outcome. One study showed the importance of identifying a subcortical language tract that is associated with postoperative language deficits [4], another study revealed the relationship between a relapse in naming and temporal and frontal tumours, and a decrease in attentional matrices in patients harbouring frontal tumours [49]. Removal of a glioma in the uncinate fasciculus related to deterioration in famous face naming [50] and glioma resection in language areas was associated with a decline in language and the executive functions [61]. Insular tumour patients performed worse on a naming test (and slightly on memory) than other tumour patients [71].

**Table 4:** Localisation and cognitive outcome

Study	Hemisphere	Localisation	Effect on cognitive outcome
Bello et al. 2007	LH + RH	Frontal, paralimbic, parietal, temporal	Subcortical language tract associated with postoperative language deficits
Teixidor et al. 2007	LH + RH	SMA (1 right, 1 left), left premotor, left frontal operculum, insula (2 right, 1 left), left parieto-retrocentral area, left parieto-temporo-occipital junction (3 left, 2 right)	No effect
Yoshii et al. 2008	LH + RH	No detailed information	N/A
Chainay et al. 2009		(Pre)SMA, PCC, ACC, MFG, IFG	N/A
Campanella et al. 2009	LH + RH	Temporal lobe (anterior and posterior superior, middle and inferior, insular and polar area)	RH HGG deteriorates compared to HC
Talacchi et al. 2011	LH + RH	Frontal, temporal, parieto-occipital	No effect
Papagno et al. 2011	LH	Frontal UF, temporal UF	Uncinate removal in frontal or temporal lobe associated with deterioration in famous face naming T2 and on object naming at T3. Word list learning at T1-T2. TMTAB on T1-T2,
			Temporal group worse than frontal in naming objects and famous faces
Sarubbo et al. 2011	LH + RH	SMA, IFG, temporal, parietal lobe	N/A
Wu et al. 2011	LH + RH	Insula, frontal, temporal, parietal, insula	Worse naming performance in insular tumours
			Trend more decline in learning and memory in insular gliomas
Matavelli et al. 2012	LH	Medial and lateral frontal lobe	No effect
Papagno et al. 2012	LH + RH	Frontal, temporal, parietal	Temporal (LH) glioma associated with relapse in naming (face + object), verbal fluency
			Frontal (LH) glioma associated with relapse in attentional matrices
			Frontal and temporal (LH) associated with relapse in object naming
Zhao et al. 2012	Dominant hemisphere	AF in frontal, precentral, temporal, insular lobe	N/A
Santini et al. 2012	Dominant hemisphere	Frontal, parietal, temporal	N/A
Satoer et al. 2012	LH	Frontal, parietal, temporal	Localisation in “classic” language areas associated with decrease on naming and category fluency
Moritz-Gasser et al. 2012	LH + RH	SMA, IFG, R SMG, parietal temporor-insula lobe, frontal lobe (anterior), fronto-insular lobe, fronto-temporal insular lobe, temporal lobe (posterior)	N/A
Moritz-Gasser et al. 2013	LH	Temporal, temporo-occipital	N/A
Satoer et al. 2013	LH	Frontal, parietal, temporal	No effect between language and motor areas

*LH* left hemisphere, *RH* right hemisphere, *SMA* supplementary motor area, *PCC* posterior cingulated cortex, *ACC* anterior cingulated cortex, *MFG* middle frontal gyrus, *IFG* inferior frontal gyrus, *SMG* superior marginal gyrus, *UF* uncinate fasciculus

*N/A* not applicable (no analysis conducted)

Seven studies looked at tumour volume/extent of resection and cognitive outcome. Most studies did not find a relationship, apart from two who found that a larger volume was associated with worsening of language and executive functioning [49, 64]. The effect of adjuvant therapy has been studied in two papers, but they did not find a relationship [59, 61]. *See* Table 3 for specifics on cognitive outcome and tumour-related and/or treatment-related factors.

## Discussion

This systematic review provides an overview of the short-term and longer-term effects of glioma surgery on cognition assessed with standardised neuropsychological tests. If available, tumour-related and/or treatment-related risk factors were described as well. We identified 17 articles in which the short-term and/or longer-term effect of neurosurgery on cognitive functioning was discussed. Generally, direct postoperative deterioration was reported followed by either recovery or remaining deterioration in one or more domains in a further follow-up (at 3–6 months), indicating the relevance of extensive neuropsychological testing. However, not all studies were representative regarding the conclusive effects of glioma surgery on cognitive functioning. For instance, test-batteries did not always cover all cognitive domains, statistical comparisons between available test-moments were not consistently conducted, follow-up range across patients was too wide, or follow-up time was not precisely described. A number of recommendations will be provided for future studies focusing on cognitive outcome after awake surgery.

### Test protocol and procedure

In order to investigate the effect of neurosurgery on cognition, it is crucial to select a set of sensitive tests for cognitive change, as LGG patients are not heavily disturbed. The prognostic property of a subnormal naming performance for immediate postoperative aphasia was already demonstrated in primary brain tumour patients [29]. This test was the most frequently used language task and appeared, as expected, to be sensitive; both improvement and deterioration were observed. Only 6 out of 17 studies, investigated cognition thoroughly, i.e., with an extensive neuropsychological test-protocol for all domains [49, 50, 57, 61, 64, 71]. Half of the studies only focused on one or two cognitive domains, which is obviously too limited to interpret the effect of surgery on overall cognition.

A comparison between all available time measurements is necessary to obtain a complete understanding of the course of recovery. Not all studies conducted comparisons with the available data between test-moments postoperatively, e.g., between the immediate postoperative phase (T2) and follow-up moment (T3) [4, 40, 66]. Two studies did not clearly report on follow-up moments [58, 72]. Also, the follow-up range of some studies may have been too wide; i.e. 3–6 months and 6–12 months [46, 57]. Deficits at 3–4 months postoperatively are considered ‘transient, compared with ‘persistent’ at 6 months and ‘permanent’ at 12 months [63], hence, one should aim for a minimal time range as possible between test-moments across patients, not exceeding these aforementioned different recovery phases. In summary, the assessment of all cognitive domains combined with a comparison between all available test-moments with a minimal time range is necessary to obtain a valuable cognitive profile of patients.

### Effects of surgery on cognition

First, the identification of impairments at baseline level is important, as these deficits are assumed to be caused by the tumour itself. Given this information, the effects of surgery can be better clarified. Not all articles performed a statistical group comparison to a normative group on cognition before surgery. Some provided percentages of impaired tests, whereas others only used preoperative baseline scores for comparison to postoperative scores.

The general finding is that cognitive status deteriorated directly after surgery followed by improvements or a decline several months after surgery [4, 9, 45, 58, 66, 72]. In particular, in the immediate postoperative phase most studies found a deterioration in the language domain. Zhao et al. [73] was the only study reporting on a significant language improvement in the immediate postoperative phase, followed by a consecutive improvement at 3–6 months postoperatively, with a general Aphasia Quotient. It is possible that a general evaluation surpasses (subtle) language deficits at separate linguistic abilities, such as naming or verbal fluency. Language, as examined by standardised tasks and also spontaneous speech, appeared to be a dynamic domain, indicating the relevance of linguistic monitoring preoperatively and postoperatively. On the other hand, all studies examined language, which may have biased the results.

One study concluded no cognitive change in a follow-up, suggesting no negative effect of surgery [58]. The statistics (or definition of the threshold), however, were not well documented, resulting in a more descriptive status of cognition in glioma patients. They also mentioned a subnormal cognitive performance both before and after operation, suggesting no negative effect of surgery [72]. Yet, it remained unclear whether different cognitive domains were taken together and if so, in what manner.

Between the immediate postoperative phase and follow-up, three studies found a significant improvement in the domains of language, memory and attention and executive functioning. In particular with the following tests: naming, verbal fluency, verbal recall and TMTA, B. Some studies reported no difference between postoperative follow-up and preoperative performance after deterioration in the immediate postoperative phase, suggesting recovery to preoperative baseline level [4, 45, 46, 50, 57] and one study found an improvement in memory [61].

Despite these positive outcome results, a large number of studies still found remaining deterioration in the follow-up phase in the before-mentioned tasks that also showed improvements. In addition, deterioration was found in famous face naming, naming time and spontaneous speech [46, 50, 57, 59, 61, 71]. Consequently, the neuropsychological research in relation to the effects of tumour resection shows mixed results about the outcome. A definite conclusion cannot be drawn yet. A more homogeneous picture could arise, when more studies about the influence of surgery on cognition, are available, the aforementioned tests show mixed results on outcome. After the inclusion date of this systematic search, two other long-term studies by Raysi et al. [51] and Satoer et al. [60] until 1 year after surgery were published where cognitive recovery only set off after the “classic 3 months period”.

The sensitive tests for change took part of larger test-batteries. Longer protocols may have caused fatigue in patients, resulting in worse task performance. To minimise a potential intervening factor as such, it may be helpful to eliminate insensitive tasks, such as non-verbal memory and visuospatial tests revealed by this review. The insensitivity of these tests could be explained by their specificity for right-hemisphere functioning, whereas most patients harboured left-hemipheric tumours. Also some subtests from the Aachener Aphasia Test (AAT) [25], Boston Diagnostic Aphasia Examination (BDAE) [41] or Batteria per l’analisi dei deficit afasici (BADA) [42] (e.g. phonemic discrimination, writing to dictation and reading) were not sensitive, possibly because these tasks are designed to measure more severe language disturbances, as in stroke patients. Finally, intraoperative studies indicated the relevance of an adapted Stroop test in the anterior cingulate cortex [68] and calculation abilities in the left parietal lobe [54]. The use of calculation tasks was not identified by this review but should also be considered in the neuropsychological protocol.

### Tumour-related factors, adjuvant therapy and cognition

As for tumour-related factors, six studies analysed cognitive performance of solely grade II LGG patients. From clinical practice, it is known that a part of supposed LGG on magnetic resonance imaging (MRI) appears to be HGG after pathological examination. It is therefore important to consecutively analyse the entire clinical group treated for glioma surgery without excluding those with grade III or IV in retrospect and thus eliminating a bias towards cognitive outcome.

Localisation in temporal or frontal areas appeared to be important for mostly language functioning, in accordance with the known neural organisation of linguistic functions [47]. More specifically, patients with tumours located in the proximity of subcortical language tracts, such as the uncinate fasciculus, were more at risk for postoperative language disturbances [4, 50], in contrast to patients with tumours nearby the arcuate fasciculus (AF) [73]. It is possible that preservation of AF with direct electrocortical stimulation results in better prognosis, as the AF was found to be predictive for overall efficiency of speech and naming in stroke patients [38]. However, most studies that were included in this review collapsed different eloquent areas in left and/or right hemisphere in their analysis, i.e. frontal, parietal, occipital, temporal and/or insular. As it was recently thoroughly described by Coello et al. [11], different (sub)cortical brain areas are associated with different functions, such as calculation in the left angular gyrus or semantic association in the temporal lobe. Tumours in language areas may have different impacts on outcome than tumours in the parietal lobe or adjacent to language areas. For tumours in language areas, De Witte et al. [18] provided guidelines for specific linguistic tasks based on tumour location. It is advisable to select cognitive tests in the preoperatively and postoperative phase based on tumour location.

Adverse effects on cognition by adjuvant therapy (radio/chemo) were not found in this review, mainly because the goal concerned investigating the effects of neurosurgery. It is known that radiotherapy and/or chemotherapy can affect cognitive functioning [2, 65], articles discussing this matter, however, were excluded due to the absence of a preoperative test-moment. Several studies showed that a negative effect on cognitive performance was absent until several years after treatment [32, 65]. The longest follow-up period in our review concerned 6 months. We therefore expect minimal to no cognitive decline associated with the use of adjuvant therapy.

### Limitations

Although we homogeneously selected the included studies based on preoperative and postoperative neuropsychological testing, this review underlines the need for more consistent neuropsychological research in glioma patients as a number of heterogeneous factors may have interfered with our results: (1) Bias to the language domain. Not all studies conducted tests covering all cognitive domains; some studies found no differences between preoperative and long-term postoperative neuropsychological assessment. These studies focused on the language domain. However, it is possible that deterioration occurred in a different domain for those patients in which language improved. (2) Test interval. Test intervals following resection varied across studies and eloquent areas were not always defined in a similar way. (3) Tumour location. Some included mixed eloquent areas, whereas others included patients with specific tumour location.

In addition, most articles discussed patients with tumour in the left hemisphere. The importance of tasks for the right-hemispere should not be ignored. Charras et al. [10] underlined the sensitivity of a line bisection task for tumours in the right angular gyrus. Also, mentalisation tasks (social cognition) with, for instance, the “Reading the Mind in the Eyes” task appeared to be relevant for lesions in the right pars opercularis and the dorsal part of the right pars triangularis [27].

However, if we would have used the abovementioned reasons as exclusion criteria, only a few studies would be selected for inclusion in this literature study. The main goal of this review concerned providing an overview of the current state of affairs on cognitive examination in consecutive glioma patients. For the design of a neuropsychological outcome study for glioma patients, one should avoid the inconsistencies across studies as described above.

No inter-observer analysis was conducted on the selected articles due to the large number of retrieved articles (*n* = 3,133). However, in case of doubt, extensive discussion was carried out between authors.

Finally, it is unclear whether cognitive rehabilitation may have influenced postoperative cognitive performance. There are some studies who demonstrated a positive effect of a computerised cognitive training program on (subjective and objective) (visual) attention and verbal memory [24, 74] short and longer term after surgery. For language, the effect of rehabilitation remains unknown in glioma patients and deserves further attention. In the stroke literature, results of speech and language treatment are defined as promising, but not evident [7]. As the results on cognitive outcome (attention, verbal memory and language) from the selected studies are ambiguous and the application of therapy was mostly not reported, it is difficult to speculate about (possible) effects.

### Recommendations for cognitive testing

In summary, some important recommendations for cognitive testing arose from this literature review:

- Test-moments: preoperative testing to define baseline deficits, postoperative follow-up testing at 3 months, 6 months and 12 months with minimal time interval.
- Neuropsychological tests-protocol for tumours in eloquent areas should at least consist of: language: naming, category and letter fluency, memory: word learning test, attention/executive functioning: TMT A and B.
- Add more specific tests for certain tumour locations: such as famous face naming for tumours in the left uncinate fasciculus (frontal and temporal), calculation in the left angular gyrus. For extensive linguistic testing for the left dominant hemisphere, *see* De Witte et al. [18]. For the right-hemisphere, the following tasks should be taken into consideration: (adapted) Stroop test in the right anterior cingulate cortex, visuo-spatial tasks in the right angular gryus and mentalisation tasks in the right pars opercularis and the dorsal part of the right pars triangularis.

## Conclusions

This review article has provided an important overview of the sensitivity of cognitive tasks as well as the course of recovery in cognition after glioma surgery. Although many studies reported recovery of cognitive function(s) after glioma surgery to the preoperative level, the more extensive neuropsychological protocols still found deterioration in some cognitive domains in a follow-up, indicating the necessity for the administration of several tasks in different cognitive domains. From these results, we can derive that one should be cautious with the general assumption of full recovery within 3 months after surgery of cognitive functions. Distinct results on outcome in the follow-up phase demand more research with larger patient groups to better understand the consequences of surgery on cognition. The standard neuropsychological test protocol should at least consist of the revealed sensitive tasks, i.e. object naming, verbal fluency, verbal word learning and Trailmaking Test B. The language domain appeared to be the most dynamic with standardised tasks, latency effects (naming time) and spontaneous speech. This suggests that intraoperative language testing at different levels should be carefully conducted, which may lead to less severe postoperative language disturbances. In conclusion, we demonstrated that cognitive recovery, with the focus on language, to preoperative baseline level is possible to a certain extent, but that the results are still arbitrary to draw definite conclusions. Most outcome results were based on a follow-up of 3–6 months. More prospective follow-up studies exceeding this period investigating all cognitive domains with the sensitive tasks for change are crucial to elucidate the long-term effects of glioma surgery.

## Appendix I: search string embase

(cognition/exp OR ‘cognitive defect’/exp OR ‘speech and language’/exp OR ‘speech disorder’/exp OR ‘language disability’/exp OR memory/exp OR neuropsychology/de OR ‘mental function’/de OR ‘intellectual impairment’/exp OR (((function* OR execut* OR psycholinguistic OR linguistic OR brain OR intellect* OR intellig* OR verbal* OR mental) NEAR/3 (control OR dysfunction* OR disabil* OR impair* OR defec* OR disturb* OR problem* OR difficult* OR disorder* OR performance* OR abilit* OR capabilit* OR capacit* OR competenc* OR outcome* OR assessment* OR evaluat* OR examin* OR monitor*)) OR ((execut* OR psycholinguistic OR linguistic OR brain OR intellect* OR intellig* OR verbal* OR mental) NEAR/3 function*) OR cognit* OR speech OR languag* OR articulat* OR aphas* OR memor* OR attention* OR alertness* OR awareness* OR concentrat* OR neurocognit* OR neuropsycholog*):ab,ti) AND (glioma/de OR oligodendroglioma/de OR astrocytoma/exp OR (astrogliom* OR oligoastrocytom* OR gliom* OR oligodendrogliom* OR astrocytom* OR (supratentorial NEXT/1 lesion*)):ab,ti) AND (surgery/exp OR surgery:lnk OR ‘postoperative complication’/exp OR (surger* OR surgic* OR craniotom* OR postoperat* OR postsurg* OR preoperat* OR presurg* OR operati*):ab,ti) NOT (‘child’/exp OR ‘childhood’/exp OR ‘childhood disease’/exp OR ‘newborn’/exp OR ‘adolescent’/exp OR ‘adolescence’/exp NOT (‘adult’/exp OR ‘adulthood’/exp OR ‘aged’/exp OR ‘middle aged’/exp)) AND [english]/lim NOT ([conference abstract]/lim OR [conference paper]/lim OR [conference review]/lim OR [editorial]/lim OR [erratum]/lim OR [letter]/lim OR [note]/lim OR [review]/lim)

## References

[1] Archibald YM, Lunn D, Ruttan LA, Macdonald DR, Del Maestro RF, Barr HWK, Pexman JHW, Fisher BJ, Gaspar LE, Cairncross JG (1994). Cognitive functioning in long-term survivors of high-grade glioma. J Neurosurg.

[2] Armstrong CL, Hunter JV, Ledakis GE, Cohen B, Tallent EM, Goldstein BH, Tochner Z, Lustig R, Judy KD, Pruitt A, Mollman JE, Stanczak EM, Jo MY, Than TL, Phillips P (2002). Late cognitive and radiographic changes related to radiotherapy: initial prospective findings. Neurology.

[3] Bartha L, Knosp E, Pfisterer W, Benke T (2000). Intra- and perioperative monitoring of language functions in patients with tumours in the left perisylvian area. Aphasiolog.

[4] Bello L, Gallucci M, Fava M, Carrabba G, Giussani C, Acerbi F, Baratta P, Songa V, Conte V, Branca V, Stocchetti N, Papagno C, Gaini SM (2007). Intraoperative subcortical language tract mapping guides surgical removal of gliomas involving speech areas. Neurosurgery.

[5] Berger MS (1994). Lesions in functional (“eloquent”) cortex and subcortical white matter. Clin Neurosurg.

[6] Bizzi A, Nava S, Ferre F, Castelli G, Aquino D, Ciaraffa F, Broggi G, DiMeco F, Piacentini S (2012). Aphasia induced by gliomas growing in the ventrolateral frontal region: assessment with diffusion MR tractography, functional MR imaging and neuropsychology. Cortex.

[7] Brady MC, Kelly H, Godwin J, Enderby P (2012) Speech and language therapy for aphasia following stroke. Cochrane Datab Syst Rev 5:CD000425 10.1002/14651858.CD000425.pub2 20464716

[8] Campanella F, Mondani M, Skrap M, Shallice T (2009). Semantic access dysphasia resulting from left temporal lobe tumours. Brain.

[9] Chainay H, Francois-Xaxier A, Alexandre K, Hugues D, Laurent C, Emmanuelle V, Laurent C, Stephane L (2009). Motor and language deficits before and after surgical resection of mesial frontal tumour. Clin Neurol Neurosurg.

[10] Charras P, Herbet G, Deverdun J, de Champfleur NM, Duffau H, Bartolomeo P, Bonnetblanc F (2015). Functional reorganization of the attentional networks in low-grade glioma patients: a longitudinal study. Cortex.

[11] Coello AF, Moritz-Gasser S, Martino J, Martinoni M, Matsuda R, Duffau H (2013) Selection of intraoperative tasks for awake mapping based on relationships between tumor location and functional networks. J Neurosurg 119:1380–1394 10.3171/2013.6.JNS122470 24053503

[12] Correa DD, DeAngelis LM, Shi WTH, Lin M, Le A (2007). Cognitive functions in low-grade gliomas: disease and treatment effects. J Neurooncol.

[13] Correa DD, Shi W, Thaler HT, Cheung AM, DeAngelis LM, Le A (2008). Longitudinal cognitive follow-up in low grade gliomas. J Neurooncol.

[14] De Benedictis A, Moritz-Gasser S, Duffau H (2010). Awake mapping optimizes the extent of resection for low-grade gliomas in eloquent areas. Neurosurgery.

[15] De Groot M, Douw L, Sizoo EM, Bosma I, Froklage FE, Heimans JJ, Postma TJ, Klein M, Reijneveld JC (2013). Levetiracetam improves verbal memory in high-grade glioma patients. Neuro Oncol.

[16] De Witt Hamer PC, Robles SG, Zwinderman AH, Duffau H, Berger MS. (2012) Impact of intraoperative stimulation brain mapping on glioma surgery outcome: a meta-analysis. J Clin Oncol 30:2559–2565 10.1200/JCO.2011.38.4818 22529254

[17] De Witte E, Marien P (2013). The neurolinguistic approach to awake surgery reviewed. Clin Neurol Neurosurg.

[18] De Witte E, Satoer D, Robert E, Colle H, Verheyen S, Visch-Brink E, Marien P (2015). The Dutch Linguistic Intraoperative Protocol: a valid linguistic approach to awake brain surgery. Brain Lang.

[19] Duffau H, Bauchet L, Lehericy S, Capelle L (2001). Functional compensation of the left dominant insula for language. Neuroreport.

[20] Duffau H, Capelle L, Denvil D, Sichez N, Gatignol P, Taillandier L, Lopes M, Mitchell MC, Roche S, Muller JC, Bitar A, Sichez JP, Van Effenterre R (2003). Usefulness of intraoperative electrical subcortical mapping during surgery for low-grade gliomas located within eloquent brain regions: functional results in a consecutive series of 103 patients. J Neurosurg.

[21] Duffau H, Denvil D, Lopes M, Gasparini F, Cohen L, Capelle L, Van Effenterre R (2002). Intraoperative mapping of the cortical areas involved in multiplication and subtraction: an electrostimulation study in a patient with a left parietal glioma. J Neurol Neurosurg Psychiatry.

[22] Duffau H, Lopes M, Arthuis F, Bitar A, Sichez JP, Van Effenterre R, Capelle L (2005). Contribution of intraoperative electrical stimulations in surgery of low grade gliomas: a comparative study between two series without (1985–96) and with (1996–2003) functional mapping in the same institution. J Neurol Neurosurg Psychiatry.

[23] Ebel H, Ebel M, Schillinger G, Klimek M, Sobesky J, Klug N (2000). Surgery of intrinsic cerebral neoplasms in eloquent areas under local anesthesia. Minim Invasive Neurosurg.

[24] Gehring K, Sitskoorn MM, Gundy CM, Sikkes SA, Klein M, Postma TJ, van den Bent MJ, Beute GN, Enting RH, Kappelle AC, Boogerd W, Veninga T, Twijnstra A, Boerman DH, Taphoorn MJ, Aaronson NK (2009). Cognitive rehabilitation in patients with gliomas: a randomized, controlled trial. J Clin Oncol.

[25] Graetz S, De Blesser R, Willmes K (1991) Akense Afasie test, Dutch edn. Swets & Zeitlinger, Lisse

[26] Hahn CA, Dunn RH, Logue PE, King JH, Edwards CL, Halperin EC (2003). Prospective study of neuropsychologic testing and quality-of-life assessment of adults with primary malignant brain tumors. Int J Radiat Oncol Biol Phys.

[27] Herbet G, Lafargue G, Moritz-Gasser S, Bonnetblanc F, Duffau H (2015). Interfering with the neural activity of mirror-related frontal areas impairs mentalistic inferences. Brain Struct Funct.

[28] Houben M, Aben K, Teepen J, Schouten-Van Meeteren A, Tijssen C, Van Duijn C, Coebergh J (2006). Stable incidence of childhood and adult glioma in The Netherlands. Acta Oncol.

[29] Ilmberger J, Ruge M, Kreth FW, Briegel J, Reulen HJ, Tonn JC (2008). Intraoperative mapping of language functions: a longitudinal neurolinguistic analysis. J Neurosurg.

[30] Johnson DR, Sawyer AM, Meyers CA, O’Neill BP, Wefel JS (2012). Early measures of cognitive function predict survival in patients with newly diagnosed glioblastoma. Neuro Oncol.

[31] Klein M, Duffau H, Hamer PCD (2012). Cognition and resective surgery for diffuse infiltrative glioma: an overview. J Neurooncol.

[32] Klein M, Heimans JJ, Aaronson NK, Van Der Ploeg HM, Grit J, Muller M, Postma TJ, Mooij JJ, Boerman RH, Beute GN, Ossenkoppele GJ, Van Imhoff GW, Dekker AW, Jolles J, Slotman BJ, Struikmans H, Taphoorn MJB (2002). Effect of radiotherapy and other treatment-related factors on mid-term to long-term cognitive sequelae in low-grade gliomas: a comparative study. Lancet.

[33] Lilja AM, Portin RI, Hamalainen PI, Salminen EK (2001). Short-term effects of radiotherapy on attention and memory performances in patients with brain tumors. Cancer.

[34] Lubrano V, Draper L, Roux FE (2010). What makes surgical tumor resection feasible in Broca’s area? Insights into intraoperative brain mapping. Neurosurgery.

[35] Maldonado IL, Moritz-Gasser S, De Champfleur NM, Bertram L, Moulinie G, Duffau H (2011). Surgery for gliomas involving the left inferior parietal lobule: new insights into the functional anatomy provided by stimulation mapping in awake patients: clinical article. J Neurosurg.

[36] Mandonnet E, Delattre JY, Tanguy ML, Swanson KR, Carpentier AF, Duffau H, Cornu P, Van Effenterre R, Alvord EC Jr, Capelle L (2003) Continuous growth of mean tumor diameter in a subset of grade II gliomas. Ann Neurol 53:524–528 10.1002/ana.10528 12666121

[37] Mandonnet E, Delattre JY, Tanguy ML, Swanson KR, Carpentier AF, Duffau H, Cornu P, Van Effenterre R, Alvord EC, Capelle L (2003). Continuous growth of mean tumor diameter in a subset of grade II gliomas. Ann Neurol.

[38] Marchina S, Zhu LL, Norton A, Zipse L, Wan CY, Schlaug G (2011) Impairment of speech production predicted by lesion load of the left arcuate fasciculus. Stroke 42:2251–2256 10.1161/STROKEAHA.110.606103 PMC3167233 21719773

[39] Marson DC, Martin RC, Triebel KL, Nabors LB (2010). Capacity to consent to research participation in adults with malignant glioma. J Clin Oncol.

[40] Mattavelli G, Casarotti A, Forgiarini M, Riva M, Bello L, Papagno C (2012). Decision-making abilities in patients with frontal low-grade glioma. J Neurooncol.

[41] Mazaux JM, Orgogozo JM (1982) Echelle d’évaluation de l’aphasie adaptée du Boston diagnostic aphasia examination. EAP Editions Psychotechniques, Paris

[42] Miceli G, Laudanna C, Burani C, Papasso R (1994) Batteria per l’analisi dei deficit afasici (BADA). Universita Cattolica del Sacro Cuore Policlinico Gemelli/Cepsag, Rome

[43] Miceli G, Capasso R, Monti A, Santini B, Talacchi A (2012). Language testing in brain tumor patients. J Neurooncol.

[44] Miotto EC, Junior AS, Silva CC, Cabrera HN, Machado MAR, Benute GRG, Lucia MCS, Scaff M, Teixeira MJ (2011). Cognitive impairments in patients with low grade gliomas and high grade gliomas. Arq Neuropsiquiatr.

[45] Moritz-Gasser S, Herbet G, Duffau H (2013) Mapping the connectivity underlying multimodal (verbal and non-verbal) semantic processing: a brain electrostimulation study. Neuropsychologia 51:1814–1822 10.1016/j.neuropsychologia.2013.06.007 23778263

[46] Moritz-Gasser S, Herbet G, Maldonado IL, Duffau H (2012). Lexical access speed is significantly correlated with the return to professional activities after awake surgery for low-grade gliomas. J Neurooncol.

[47] Naidich T, Hof P, Gannon P, Yousry T, Yousry I (2001) Anatomic substrates of language: emphasizing speech. Neuroimag Clin North Am II:305–341 11489742

[48] Ojemann JG, Miller JW, Silbergeld DL (1996). Preserved function in brain invaded by tumor. Neurosurgery.

[49] Papagno C, Casarotti A, Comi A, Gallucci M, Riva M, Bello L (2012). Measuring clinical outcomes in neuro-oncology. A battery to evaluate low-grade gliomas (LGG). J Neurooncol.

[50] Papagno C, Miracapillo C, Casarotti A, Romero Lauro LJ, Castellano A, Falini A, Casaceli G, Fava E, Bello L (2011). What is the role of the uncinate fasciculus? Surgical removal and proper name retrieval. Brain.

[51] Raysi Dehcordi S, Mariano M, Mazza M, Galzio RJ (2013). Cognitive deficits in patients with low and high grade gliomas. J Neurosurg Sci.

[52] Reijneveld JC, Sitskoorn MM, Klein M, Nuyen J, Taphoorn MJB (2001). Cognitive status and quality of life in patients with suspected versus proven low-grade gliomas. Neurology.

[53] Robles SG, Gatignol P, Capelle L, Mitchell MC, Duffau H (2005). The role of dominant striatum in language: a study using intraoperative electrical stimulations. J Neurol Neurosurg Psychiatry.

[54] Roux F-E, Boetto S, Sacko O, Chollet F, Trémoulet M (2003). Writing, calculating, and finger recognition in the region of the angular gyrus: a cortical stimulation study of Gerstmann syndrome. J Neurosurg.

[55] Sakurada K, Sato S, Sonoda Y, Kokubo Y, Saito S, Kayama T (2007). Surgical resection of tumors located in subcortex of language area. Acta Neurochir.

[56] Sanai N, Mirzadeh Z, Berger MS (2008). Functional outcome after language mapping for glioma resection. New Engl J Med.

[57] Santini B, Talacchi A, Squintani G, Casagrande F, Capasso R, Miceli G (2012). Cognitive outcome after awake surgery for tumors in language areas. J Neurooncol.

[58] Sarubbo S, Latini F, Panajia A, Candela C, Quatrale R, Milani P, Fainardi E, Granieri E, Trapella G, Tugnoli V, Cavallo MA (2011). Awake surgery in low-grade gliomas harboring eloquent areas: 3-year mean follow-up. Neurol Sci.

[59] Satoer D, Vincent A, Smits M, Dirven C, Visch-Brink E (2013). Spontaneous speech of patients with gliomas in eloquent areas before and early after surgery. Acta Neurochir.

[60] Satoer D, Visch-Brink E, Smits M, Kloet A, Looman C, Dirven C, Vincent A (2014). Long-term evaluation of cognition after glioma surgery in eloquent areas. J Neurooncol.

[61] Satoer D, Vork J, Visch-Brink E, Smits M, Dirven C, Vincent A (2012). Cognitive functioning early after surgery of gliomas in eloquent areas. J Neurosurg.

[62] Skirboll SS, Ojemann GA, Berger MS, Lettich E, Winn HR (1996). Functional cortex and subcortical white matter located within gliomas. Neurosurgery.

[63] Talacchi A, D’Avella D, Denaro L, Santini B, Meneghelli P, Savazzi S, Gerosa M (2012). Cognitive outcome as part and parcel of clinical outcome in brain tumor surgery. J Neurooncol.

[64] Talacchi A, Santini B, Savazzi S, Gerosa M (2011). Cognitive effects of tumour and surgical treatment in glioma patients. J Neurooncol.

[65] Taphoorn MJB, Schiphorst AK, Snoek FJ, Lindeboom J, Wolbers JG, Karim ABMF, Huijgens PC, Heimans JJ (1994). Cognitive functions and quality of life in patients with low-grade gliomas: the impact of radiotherapy. Ann Neurol.

[66] Teixidor P, Gatignol P, Leroy M, Masuet-Aumatell C, Capelle L, Duffau H (2007). Assessment of verbal working memory before and after surgery for low-grade glioma. J Neurooncol.

[67] Thomson AM, Taylor R, Fraser D, Whittle IR (1997). Stereotactic biopsy of nonpolar tumors in the dominant hemisphere: a prospective study of effects on language functions. J Neurosurg.

[68] Wager M, Du Boisgueheneuc F, Pluchon C, Bouyer C, Stal V, Bataille B, Guillevin CM, Gil R (2013) Intraoperative monitoring of an aspect of executive functions: administration of the Stroop test in 9 adult patients during awake surgery for resection of frontal glioma. Neurosurg 72:ons169–180; discussion ons180–161 10.1227/NEU.0b013e31827bf1d6 23149965

[69] Whittle IR, Borthwick S, Haq N (2003). Brain dysfunction following ‘awake’ craniotomy, brain mapping and resection of glioma. Br J Neurosurg.

[70] Whittle IR, Pringle AM, Taylor R (1998). Effects of resective surgery for left-sided intracranial tumours on language function: a prospective study. Lancet.

[71] Wu AS, Witgert ME, Lang FF, Xiao L, Bekele BN, Meyers CA, Ferson D, Wefel JS (2011). Neurocognitive function before and after surgery for insular gliomas: clinical article. J Neurosurg.

[72] Yoshii Y, Tominaga D, Sugimoto K, Tsuchida Y, Hyodo A, Yonaha H, Kushi S (2008). Cognitive function of patients with brain tumor in pre- and postoperative stage. Surg Neurol.

[73] Zhao Y, Chen X, Wang F, Sun G, Wang Y, Song Z, Xu B (2012). Integration of diffusion tensor-based arcuate fasciculus fibre navigation and intraoperative MRI into glioma surgery. J Clin Neurosci.

[74] Zucchella C, Capone A, Codella V, De Nunzio AM, Vecchione C, Sandrini G, Pace A, Pierelli F, Bartolo M (2013). Cognitive rehabilitation for early post-surgery inpatients affected by primary brain tumor: a randomized, controlled trial. J Neurooncol.

